# The impact of risk information frameworks on cancer drug insurance (CDI) purchase decisions through time orientation and perceived risk: a survey-experiment study

**DOI:** 10.3389/fpubh.2026.1757999

**Published:** 2026-02-05

**Authors:** Zhenyu Sun, Muhammad Aditya Kurnia, Ziying Zhang, Yue Wang, Dongfu Qian

**Affiliations:** 1 School of Health Policy & Management, Nanjing Medical University, Nanjing, China; 2 Laboratory for Digital Intelligence & Health Governance, Nanjing Medical University, Nanjing, China

**Keywords:** cancer drug insurance, China, perceived risk, risk information framework, time orientation

## Abstract

**Background:**

China is actively promoting cancer drugs insurance (CDI) to alleviate the growing burden of cancer. However, the efficacy of risk information frameworks supplied to consumers in expanding CDI coverage remains poorly understood.

**Purpose:**

This study aimed to examine the impact of the risk information framework (RIF) on individuals’ CDI purchase decisions, as well as the roles played by time orientation and perceived risk within these frameworks.

**Methods:**

This nationwide online survey enrolled 5,583 eligible participants aged from 18 to 60 years old in August 2025 in China. A randomized survey-based experiment with the designed risk information frameworks was conducted to elicit CDI purchase decisions. Participants were exposed to either a low-risk information framework (LRIF) or a high-risk information framework (HRIF) before making CDI purchase decisions. Descriptive statistics and mediating effect models were used to analyze participants’ decisions to purchase CDI.

**Results:**

Among 2,825 eligible participants in LRIF, 364 (12.9%) were not insured by CDI, and 1,507 (53.3%) were insured by long-term CDI schemes (selected the option of 3 years). Among 2,758 eligible participants in HRIF, 99 (3.6%) were not insured by CDI, and 1881 (68.2%) were insured by long-term CDI schemes. Under LRIF, the mediating effects of perceived risk between present-oriented attitude and CDI purchase were −0.030 [95%CI: −0.072, −0.003], and between future-oriented attitude and CDI purchase were 0.089 [95%CI: 0.064, 0.118]. Under HRIF, the corresponding mediating effects of perceived risk were −0.006 [95%CI: −0.024, −0.001] and 0.022 [95%CI: 0.010, 0.038].

**Conclusion:**

HRIF could stimulate an individual’s CDI adoption. The underlying mechanism may be that HRIF restrains a present-oriented attitude and reinforces a future-oriented one, with perceived risk as a mediating factor. These findings underscore the need to emphasize RIF designs to promote CDI coverage.

## Introduction

Cancer constitutes a significant portion of the global disease burden (1), and has been the leading cause of death in China from 2000 to 2020 (2). In 2020 alone, approximately 2.4 million Chinese residents succumbed to cancer (3), with the economic cost estimated to reach USD 6.1 trillion nationally from 2020 to 2050 (4). While specific anticancer drugs can significantly reduce mortality and tumor progression, their exorbitant prices pose a significant barrier (5). According to Allied Market Research (AMR), global spending on cancer drugs was approximately USD 135.5 billion in 2020, projected to reach USD 274.4 billion by 2030 (6). In China, domestic targeted anticancer drugs often exceed USD 3,786 per month (7), rendering them unaffordable for most families (8).

The escalating costs of anticancer drugs have garnered international attention (9). However, the public health insurance system does not adequately shield individual cancer patients from financial toxicity (10–12). Incentivizing specialty drug coverage in commercial health plans may increase access to tumor treatments for patients (10, 11), while cancer drug insurance (CDI) coverage in China remains inadequate. Disparities in health insurance participation were ongoing concerns, with social psychologists and behavioral economists positing that perceptions and attitudes toward time and risk may explain these disparities (13, 14), and leveraging the frame effect of information presentation in promotional strategies is considered an effective tool to influence health insurance behavior (15).

In the context of China’s burgeoning “Internet plus” development (16), online sales are catalyzing the rise of CDI and garnering increased attention in recent years. Nevertheless, as observed for common online CDI advertisements (see Appendix A), we found that a rough narrative of cancer-related risk information and the same risk information frame (RIF) for different CDI schemes is a universal phenomenon in CDI advertising. We refer to rough narratives as a low-risk information framework (LRIF) and meticulous narratives as a high-risk information framework (HRIF). Considering these factors together, it is reasonable to speculate that inefficiently linking advertising to risk information is a significant factor contributing to the weakened CDI adoption in China. However, we have not found studies confirming this.

Although existing studies offer initial insights into the correlation among perceived risk, time orientation, and the use of RIF to promote health behaviors, most rely on retrospective data or laboratory experiments with student participants, yielding inconclusive evidence on the potential efficacy of RIF in health policy-making. Undoubtedly, effective debiasing strategies could be applied to gain causal understanding of factors contributing to bias, and online survey experiments may counteract biased data associated with health-related decision-making (17). However, the efficacy of the RIF provided to consumers in expanding CDI coverage remains poorly understood.

Accordingly, this study provides a contextualized simulation aligned with emerging CDI online sales advertising, aiming to understand the unique ability of RIF in shaping individuals’ decisions to purchase CDI through perceived risk and time orientation. Such research has powerful implications for developing health policies that promote CDI adoption, an emerging and prominent insurance product in China. Unless specified otherwise, the CDI discussed in the present study is commercial.

### Risk information framework

The information framework refers to how individuals respond differently to positive or negative descriptions of information (18). Two prominent models (risk and gain-loss framed) propose that the titer effects of the information framework are moderated by framing methods and perceived risk (19, 20). Risky and temporal framing are the two common formats for framing information or advertising in the health domain (21). There is evidence that the way information is framed influences decision-making regarding a variety of health behaviors (15, 22, 23). For example, results from a controlled experiment on temporal framing effects in HPV vaccination indicated that a present-oriented message presented in a narrative format led to more favorable attitudes, stronger intentions, and perceived efficacy towards the vaccine (23). Exposure to narrative messages was also found to be positively associated with intentions to discuss influenza vaccination (22).

Many theories could be used to interpret the effectiveness of risk information frameworks and how they work. Prospect theory (PT), temporal discounting theory (TDT), and construal level theory (CLT) mainly in this regard. PT indicates that health-relevant information presented in terms of value and risk can shape individual perceptions to motivate healthy behavior (24). TDT treats the reduction in the value of an intensifier as a function of its receiver delay, revealing the psychological phenomenon that an individual’s value assessment for events decreases over time (25). CLT proposes the concept of temporal construal, suggesting that the time-distance effect arises from an active network of mental representations towards future objects, predicting that feasibility concerns should receive less weight as time distance increases (26). Consequently, we observe a close relationship between RIF, perceived risk, and time orientation.

### Time orientation

Time orientation refers to how individuals value distant outcomes relative to present ones (27). Evidence from CLT suggests that health messages linked to temporal framing effects could influence individuals’ time orientation towards hazards and losses in health, and temporal framing effects in health advertising have also been found to affect future orientation on perceived risk and behavioral intentions (21, 28). The latest findings from a multilevel meta-analysis on temporal framing effects showed that gain (versus loss) framing was a significant moderator of temporal influence on promoting healthy eating and anti-smoking/drinking behaviors, and proximal frames were more effective at increasing risk perception than distal frames (29). Nevertheless, although the literature indicates a close connection between perceived risk and time orientation, some scholars do not believe that framing risk information would systematically change time orientation (21).

Time orientation with systematic differences distinguishes individuals who stress-emphasize immediate versus delayed consequences (present-orientation versus future-orientation) of their actions, and individuals with different time orientations show heterogeneity in health-related behaviors (30). An online experiment revealed that stronger present-orientation predicted greater smoking intentions, while future-orientation predicted greater quit intentions, and in addition, future (versus present) thinking significantly improved intentions to give up smoking by enhancing perceived self-efficacy for cessation (31). Given that CDI purchase decisions involve trade-offs between delayed benefits (relief from financial burden if cancer is found, or a sense of security during the guarantee period) and immediate costs (premium costs, monetary loss aversion), individuals’ weighting of long-term versus short-term consequences may influence their decisions. Therefore, future-oriented individuals may place a higher value on health insurance benefits than present-oriented individuals (32).

### Perceived risk

The weighting of benefits and costs in insurance decision-making may also depend on individuals’ perceptions and attitudes toward the risks associated with cancer (33). Perceived risk arises from uncertainty about carcinogenic factors and the economic burden before cancer diagnosis (34). Individual participant assessments of their risk and recovery probabilities from health and financial trauma caused by cancer development seem pivotal in enrolling in CDI. Theoretical models examining the correlation between perceived risk and medical insurance purchasing yield mixed predictions. There is evidence that perceived risk clearly pushes the average willingness to pay for health insurance below the fair price (35). However, there is also evidence that perceived risk related to the chances of getting cancer is not associated with health behaviors like cancer exams (36).

Researchers are committed to deepening our understanding of the driving mechanism of perceived risk and exploring how to improve the public’s risk identification ability and decision-making quality through efficient risk communication. Existing research indicates that message content framing in combination with health-related risk communications can have significant and measurable effects on consumer cognition, emotion, and behavior (37). Additionally, some analyses have shown that perceived risk plays a mediating role in the influence of online food safety information acquisition on food risk prevention behaviors (38). This potentially important framing technique, which has not received enough attention, is the differentiated risk narratives in the context of online sales advertising for CDI adoption.

Based on the literature review mentioned above, our theoretical framework posits that the RIFs (including LRIF and HRIF) serve as the intervention, time orientation serves as the independent variable, perceived risk serves as the mediating variable, CDI purchase decisions serve as the dependent variable, and we propose the following basic hypotheses regarding the RIF, perceived risk, and time orientation.

*H1*: HRIF would facilitate an individual's CDI purchase decisions for insuring or insuring long-term schemes.

*H2*: HRIF would strengthen an individual's future orientation and weaken present orientation regarding CDI purchase.

*H3*: HRIF would increase an individual's perceived risk of developing cancer.

*H4*: For facilitating an individual's CDI purchase decisions, HRIF would restrain a present-oriented attitude and reinforce a future-oriented attitude, with the mediating role of perceived risk.

Notes: LRIF refers to the use of rough narratives for risk information, while HRIF refers to the use of meticulous narratives for risk information.

## Materials and methods

### Data source

This study used a randomized nationwide online survey-based experiment, which was approved by Nanjing Medical University’s Institutional Review Board in October 2023 and was implemented by WJX (an online questionnaire survey platform owned by Changsha Ranxing Information Technology Co., Ltd. in China) in August 2025. WJX recruited contributing participants willing to engage in the survey experiment from its sample bank of 8 million people. To make participants more nationally representative, we required that the provincial and gender distributions of the participants that WJX collected were similar to the data published by the National Bureau of Statistics of China in 2022. The experiment was self-administered and accessible at any time during the designated period. The participants were allowed to complete the experiment only once and could leave at any time. Upon completing the survey, each participant received a 10 Chinese yuan (CNY) incentive from WJX to encourage thoughtful and accurate responses.

### Study participants

This study initially included a random sample of 6,044 participants aged from 18 to 60 years old, who were members of the WJX online research panel. Upon initiation of the study, participants were unaware of whether they were in the treatment (HRIF) or control (LRIF) group. The participants were informed that the survey pertained to consumption on CDI, and no precise information on the survey’s topic was provided during the recruitment process (27). After excluding invalid participants based on criteria such as minimum answer time, empty value limitation, and repeated responses, a total of 5,583 valid participants were included for analysis.

### Online survey experiment design

We conducted a randomized nationwide online survey-based experiment to explore a 2 (Part A, RIF [r]: LRIF or HRIF) × 3 (Part B, CDI schemes [j]: distinguished by free cancer drugs provided duration of 1, 2, or 3 years) factorial design on the primary outcome: participant’s choices for CDI schemes; as well as secondary outcomes about time orientation (Part C) and perceived risk (Part D) related to CDI purchase decisions, along with selected demographic characteristics (Part E) of participants. Consequently, participants were divided into groups (r.j) randomly according to the questionnaire version they responded to, which was extracted randomly from the six questionnaire versions generated based on RIF and CDI schemes. The details are as follows:

### Part a: RIF

We extracted LRIF from common online CDI advertising, which served as the control group, and simulated the current online CDI risk exposition in the market. Based on CLT in behavioral economics (23, 26, 29) and other relevant references (22, 39–41), HRIF as the treatment group was created with a much more meticulous narrative of cancer itself and its derived financial risks. The RIF is in Appendix B.

### Part B: CDI schemes

Age serves as the primary determinant for CDI online pricing. We knew the 40-something age group was the body of CDI purchase form executives at several large CDI companies. Thus, we entered age 40 as a parameter into the top 6 mainstream online CDI subscription systems and found that the average premium for insuring 1 year was 176.62 CNY. Then, the premium for just insuring 1 year was set at 179 CNY in our CDI schemes. After discounting using China’s average inflation rate (2–3%) over the past decade and the 3-year fixed deposit rate (2.20%) published by the Bank of China, the premium for insuring three consecutive years was priced at 521 CNY in our CDI schemes.

The final premium determination in our study also considered the principle of the anchoring effect in behavioral economics (42), which was to increase the premium mental calculation complexity for weakening the interference of linear price growth on the participants’ choices of CDI schemes. In this way, the decision results for the participants were more reflected in the time orientation rather than the premium change itself. The only difference between the 3 CDI schemes is the duration of free cancer drugs provided; see Appendix C.

### Part C: time orientation towards CDI adoption

To avoid order effect and propensity scoring of the participants’ responses, partially mirrored items were set and presented randomly with the WJX random question function, and the final scores of each item were displayed after the same trend processing. Time orientation was categorized into present-oriented and future-oriented attitudes (27), measured with psychometric scales, and is associated with current cancer risk and premium cost, future security, and long-term impact on life (21).

The present-oriented dimension was assessed using two items: “I focus on my current NOT future cancer risk when I choose the CDI scheme” (mirrored item: “I focus on my future NOT current cancer risk when I choose the CDI scheme”) and “I care more about current premium NOT future cost reduce when I choose the CDI scheme” (mirrored item: “I care more about future cost reduce NOT current premium when I choose the CDI scheme”).

The future-oriented dimension was evaluated using two items: “I value more the future security when I choose the CDI scheme” and “I consider its long-term impact on my future life seriously when I choose the CDI scheme.” All four items were measured on a 5-point scale ranging from 1 (disagree) to 5 (agree). Accordingly, the present-oriented and future-oriented attitudes dimensions are both scored on a scale of 2–10, with higher scores indicating a stronger corresponding time orientation.

### Part D: perceived risk related to developing cancer

Perceived risk consists of cancer and financial aspects in this study, in reference to the research (43–45). To assess the dimension of cancer, we employed 2 items focusing on the presence of carcinogenic factors and concerns about developing cancer: “Carcinogenic factors are ubiquitous in daily living and working conditions,” and “I’m worried about developing cancer in my life” (mirrored item: “I’m NOT worried about developing cancer in my life”).

To evaluate the financial dimension, we utilized 3 items concentrating on the risk of cancer drugs burden, unaffordable for family, and CDI avoids economic loss: “I’m worried about the price of cancer drugs is steep” (mirrored item: “I’m NOT worried about the price of cancer drugs is steep”), “If I get cancer, the cancer drugs expenses are unaffordable for my family” (mirrored item: “If I get cancer, the cancer drugs expenses are affordable for my family”), and “Cancer drugs insurance could avoid economic risk caused by cancer.”

All five items were measured on a 5-point scale ranging from 1 (disagree) to 5 (agree). Consequently, the perceived risk related to developing cancer was measured by summing the scores of the above two dimensions, ranging from 5 to 25. A higher score indicates a heightened level of perceived risk.

### Part E: selected demographic characteristics

Previous research has suggested that sociodemographic characteristics would potentially affect people’s health insurance purchasing decisions. We then selected seven demographic characteristics (age, gender, marital, residence, education, health insurance type, and annual disposable income individually) as control variables. It should be noted that we did not include variables such as knowledge about CDI and CDI purchase experience, as the strong information endowment of these participants may bias survey results, given that this population is more active online.

### Quality guarantee

#### Preset online survey experiment

200 undergraduates of Nanjing Medical University were selected for the pre-test. Based on their questionnaire responses and subsequent interview results, we then further polished the LRIF and HRIF narratives, refined the time orientation and perceived risk measurement items, and estimated the time required for the formal survey experiment.

#### Formal online survey experiment

First, the LRIF and HRIF interfaces were forced to stay at least 10 and 25 s, respectively, and participants could only continue the questionnaire response by clicking the “Reading Complete” button. Second, limit one IP address to one response to avoid duplicate data collection. Third, eliminate questionnaires that took less than 180 s. Fourth, excluded low-quality questionnaires with large areas (exceeding 10%) of empty values and required items with a “0” value. Finally, in accordance with the above standards, three waves of real-time quality control survey experiments were carried out (46), each round spaced 3 to 5 days apart, with around 2000 participants.

#### *Post hoc* analysis

We conducted the reliability and validity for the measurement of time orientation and perceived risk. In the mechanism analysis of CDI purchase decisions, common method variance (CMV), and endogeneity issue mainly caused by missing or unknown omitted variables (e.g., CDI purchase experience, etc.) might bias our findings, which were both tested as well. In addition, we placed the LRIF and HRIF at the bottom of the questionnaires (i.e., removed LRIF and HRIF), then took a 15% sample size and carried out the same procedure as the formal survey experiment to rule out the potential possibility of differences in results due to the RIF weakening or strengthening the original time orientation and perceived risk. All the above implemented procedures ensure the robustness of the results and conclusions in this study.

### Statistical analysis

We used a chi-square test to demonstrate the balance and comparability of valid samples across each study group, applied multiple chi-square tests to disclose the systematic differences of participants’ CDI purchase decisions, and ran an independent sample t-test to reveal the systematic differences of participants’ time orientation and perceived risk between LRIF and HRIF. Then, we conducted mediation analyses using the bootstrap method (5,000 resamples) with bias-corrected confidence intervals to test the mediating effects of perceived risk in the relationship between time orientation and CDI purchase decisions, separately within LRIF and HRIF groups. All models controlled for the selected demographic characteristics. Coefficients were reported as unstandardized estimates. Considering that the proportion mediated could be unstable when total effects were small, we emphasized the magnitude and direction of mediating effects changing from LRIF to HRIF groups.

Cronbach’s *α* coefficient (threshold is 0.6) was used to test the reliability, and exploratory factor analysis (EFA, KMO value threshold is 0.6) was employed to assess the validity. Harman’s single-factor test (threshold of 50%) was applied to evaluate CMV, and endogeneity was assessed by the Durbin–Wu–Hausman (DWH) test. All statistical analyses were performed using the R statistical software package (version 4.4.2). The *p* value of 0.05 was considered to be significant.

## Results

### Participant samples

Randomization into the six study groups yielded a total of 5,583 valid samples, demonstrating balance across selected demographic characteristics (see Table 1). This balanced distribution suggests that the impact of risk information narratives (LRIF vs. HRIF) can be examined by comparing unadjusted results across the study groups.

**Table 1:** Selected characteristics of the participants, *n* (%).

Characteristics	Group 1.1	Group 1.2	Group 1.3	Group 2.1	Group 2.2	Group 2.3	Total	*χ^2^*	*p*
Age								16.381	0.357
18 to 29 years old	416 (44.4)	441 (47.3)	426 (44.6)	437 (47.7)	442 (47.9)	455 (49.6)	2,617 (46.9)		
30 to 39 years old	344 (36.8)	342 (36.7)	334 (34.9)	313 (34.1)	323 (35.0)	300 (32.7)	1956 (35.0)		
40 to 49 years old	110 (11.8)	106 (11.4)	127 (13.3)	111 (12.1)	108 (11.7)	110 (12.0)	672 (12.0)		
50 to 60 years old	66 (7.1)	44 (4.7)	69 (7.2)	57 (6.2)	49 (5.3)	53 (5.8)	338 (6.1)		
Gender								1.446	0.919
Male	482 (51.5)	499 (53.5)	489 (51.2)	475 (51.7)	482 (52.3)	485 (52.8)	2,912 (52.2)		
Female	454 (48.5)	434 (46.5)	467 (48.8)	443 (48.3)	440 (47.7)	433 (47.2)	2,671 (47.8)		
Marital								0.872	0.972
Married	515 (55.0)	503 (53.9)	532 (55.6)	496 (54.0)	500 (54.2)	504 (54.9)	3,050 (54.6)		
Not married	421 (45.0)	430 (46.1)	424 (44.4)	422 (46.0)	422 (45.8)	414 (45.1)	2,533 (45.4)		
Residence								7.540	0.183
Rural	285 (30.4)	320 (34.3)	319 (33.4)	288 (31.4)	319 (34.6)	323 (35.2)	1854 (33.2)		
Urban	651 (69.6)	613 (65.7)	637 (66.6)	630 (68.6)	603 (65.4)	595 (64.8)	3,729 (66.8)		
Annual disposable income								13.239	0.584
Up to 19,999 CNY	208 (22.2)	220 (23.6)	241 (25.2)	213 (23.2)	227 (24.6)	226 (24.6)	1,335 (23.9)		
20,000 to 29,999 CNY	106 (11.3)	107 (11.5)	87 (9.1)	89 (9.7)	79 (8.6)	89 (9.7)	557 (10.0)		
30,000 to 49,999 CNY	400 (42.7)	386 (41.4)	394 (41.2)	416 (45.3)	403 (43.7)	385 (41.9)	2,384 (42.7)		
At least 50,000 CNY	222 (23.7)	220 (23.6)	234 (24.5)	200 (21.8)	213 (23.1)	218 (23.7)	1,307 (23.4)		
Education								19.021	0.520
Junior	24 (2.6)	27 (2.9)	33 (3.5)	26 (2.8)	39 (4.2)	30 (3.3)	179 (3.2)		
Senior	128 (13.7)	123 (13.2)	143 (15.0)	127 (13.8)	107 (11.6)	140 (15.3)	768 (13.8)		
Associate	252 (26.9)	240 (25.7)	251 (26.3)	227 (24.7)	215 (23.3)	235 (25.6)	1,420 (25.4)		
Undergraduate	488 (52.1)	492 (52.7)	491 (51.4)	494 (53.8)	517 (56.1)	472 (51.4)	2,954 (52.9)		
Postgraduate	44 (4.7)	51 (5.5)	38 (4.0)	44 (4.8)	44 (4.8)	41 (4.5)	262 (4.7)		
Health insurance type								14.703	0.793
Free	75 (8.0)	85 (9.1)	89 (9.3)	84 (9.2)	79 (8.6)	98 (10.7)	510 (9.1)		
Urban employees	408 (43.6)	379 (40.6)	396 (41.4)	375 (40.8)	367 (39.8)	366 (39.9)	2,291 (41.0)		
Urban and rural residents	397 (42.4)	409 (43.8)	410 (42.9)	408 (44.4)	419 (45.4)	389 (42.4)	2,432 (43.6)		
Commercial	41 (4.4)	35 (3.8)	42 (4.4)	30 (3.3)	37 (4.0)	38 (4.1)	223 (4.0)		
Without	15 (1.6)	25 (2.7)	19 (2.0)	21 (2.3)	20 (2.2)	27 (2.9)	127 (2.3)		
Sample size	936	933	956	918	922	918	5,583		

Group r.j, r represents the type of RIF, j represents the type of CDI schemes. r = 1 represents LRIF (control group), r = represents HRIF (treatment group). j = 1 represents CDI scheme 1, j = 2 represents CDI scheme 2, j = 3 represents CDI scheme 3. 1 CNY=US $0.14.

CDI, cancer drug insurance; RIF, risk information framework; LRIF, low risk information framework; HRIF, high risk information framework.

### Purchasing decisions for CDI schemes

Table 2 presents numbers (frequency) and their statistically significant differences of the HRIF treatment effect on CDI purchase decisions of participants compared with the LRIF control group. Under LRIF, as the period of free cancer drugs provided increased, the proportion of option 0 (not insured) and option 1 (just insured 1 year, 179 CNY premium) decreased from 18.5 to 7.5% and 40.5 to 28.2%, respectively, while the proportion of option 2 (insured 3 years consecutively, 179 CNY premium) increased from 41.0 to 64.2%.

**Table 2:** Participants’ choices of CDI schemes, *n* (%).

RIF	Duration of free cancer drugs provided after diagnosis	Group	Option 0	Option 1	Option 2	*χ^2^*	*p*
LRIF	1 year	1.1	173 (18.5)	379 (40.5)	384 (41.0)	284.736	<0.001
	2 years	1.2	119 (12.8)	305 (32.7)	509 (54.6)	46.952	<0.001
	3 years	1.3	72 (7.5)	270 (28.2)	614 (64.2)	1.110	0.574
HRIF	1 year	2.1	49 (5.3)	257 (28.0)	612 (66.7)	14.002	0.001
	2 years	2.2	35 (4.2)	228 (24.7)	655 (71.0)	38.002	<0.001
	3 years	2.3	15 (1.6)	142 (15.5)	761 (82.9)	190.085	<0.001
*χ^2^*			235.135	157.690	411.077		
*p*			<0.001	<0.001	<0.001		

Option 0 = not insured; Option 1 = just insured 1 year, 179 CNY premium; Option 2 = insured 3 years consecutively, 179 CNY premium. 1 CNY=US $0.14.

RIF, risk information framework; LRIF, low risk information frame; HRIF, high risk information frame.

Under HRIF, the proportions of options 0 and 1 decreased from 5.3 to 1.6% and 28.0 to 15.5%, respectively, while the proportion of option 2 rose from 66.7 to 82.9%. The proportion of option 0 in groups 1.1, 1.2, and 1.3 was higher than that in groups 2.1 (18.5% vs. 5.3%), 2.2 (12.8% vs. 4.2%), and 2.3 (7.5% vs. 1.6%). The proportion of option 1 in groups 1.1, 1.2, and 1.3 was higher than that in groups 2.1 (40.5% vs. 28.0%), 2.2 (32.7% vs. 24.7%), and 2.3 (28.2% vs. 15.5%). Conversely, the proportion of option 2 in groups 1.1, 1.2, and 1.3 was lower than that in groups 2.1 (41.0% vs. 66.7%), 2.2 (54.6% vs. 71.0%), and 2.3 (64.2% vs. 82.9%). Thus, H1 was verified.

### Time orientation towards CDI adoption

Table 3 displays the variance in agreement degree for present- and future-oriented attitude items expressed in LRIF and HRIF. Participants under LRIF scored on present-oriented attitude items regarding current cancer risk and premium cost were 4.49 and 4.55, respectively, which were significantly higher than those of the participants under HRIF (2.79 and 2.83). Conversely, participants under LRIF scored 3.00 and 3.14, respectively, on future-oriented attitude items related to security and long-term impact on life, which were significantly lower than those of participants under HRIF (4.69 and 4.78). Therefore, H2 was verified.

**Table 3:** Participants’ time orientations toward CDI purchase.

Time orientation	Items	LRIF (*n* = 2,825)	HRIF (*n* = 2,758)	Comparison	
		Mean (SD)	Mean (SD)	*t*	*p*
Present-oriented	I focus on my current NOT future cancer risk when I choose the CDI scheme	4.49 (0.807)	2.79 (0.986)	70.545	<0.001
	I care more about the current premium, NOT future cost reduction, when I choose the CDI scheme	4.55 (0.717)	2.83 (0.952)	76.430	<0.001
Future-oriented	I value more on the future security more when I choose the CDI scheme	3.00 (0.886)	4.69 (0.645)	81.155	<0.001
	I consider its long-term impact on my future life seriously when I choose the CDI scheme	3.14 (0.808)	4.78 (0.539)	88.905	<0.001

LRIF, low risk information frame; HRIF, high risk information frame; SD, standard deviation.

### Perceived risk related to developing cancer

Table 4 shows the difference in scores on items about perceived risk measurement for participants under LRIF and HRIF. Participants under LRIF scored points 3.18, 2.90, 2.43, 3.07, and 3.03 on the perceived risk measurement items of worrying about carcinogenic factors, developing cancer, cancer drugs burden, and unaffordability for the family. CDI avoided economic loss, which was statistically significantly lower than under the HRIF (scored in order of points 4.76, 4.58, 3.98, 4.66, and 4.67, respectively). The above results also indicated that the HRIF developed in this study was effective. Thereby, H3 was verified.

**Table 4:** Perceived risk related to developing cancer.

Perceived risk	Items	LRIF (*n* = 2,825)	HRIF (*n* = 2,758)	Comparison	
		*M* (SD)	*M* (SD)	*t*	*p*
Cancer	Carcinogenic factors are ubiquitous in daily living and working conditions	3.18 (0.872)	4.76 (0.563)	79.888	<0.001
	I’m worried about developing cancer in my life	2.90 (0.952)	4.58 (0.726)	73.944	<0.001
Financial	I’m worried that the price of cancer drugs is steep	2.43 (1.225)	3.98 (1.138)	49.017	<0.001
	If I get cancer, the cancer drug expenses are unaffordable for my family	3.07 (0.949)	4.66 (0.688)	71.675	<0.001
	Cancer drug insurance could avoid economic risk caused by cancer	3.03 (0.855)	4.67 (0.626)	81.836	<0.001

LRIF, low risk information frame; HRIF, high risk information frame; SD, standard deviation.

### Mediating mechanism of RIF intervenes on CDI purchase decisions

Table 5 shows the results of the mediation analysis examining the role of perceived risk in the relationship between time orientation and CDI purchase decisions under LRIF and HRIF, estimated via the bootstrap method. Under LRIF, the mediating effect of perceived risk between present-oriented attitude (POA) and cancer drug insurance choice (CDIC) was −0.030 [95%CI: −0.072, −0.003], and between future-oriented attitude (FOA) and CDIC was 0.089 [95%CI: 0.064, 0.118]. While under HRIF, the corresponding mediating effects of perceived risk were −0.006 [95%CI: −0.024, −0.001] and 0.022 [95%CI: 0.010, 0.038]. These results indicate that perceived risk plays a significant mediating role in the relationship between time orientation and CDI purchase decisions, and that the strength and nature of this mediation may depend on the risk information framework. Overall, H4 was validated to some extent.

**Table 5:** The mediating effect of perceived risk between time orientation and CDI purchase decisions.

Variables	LRIF		HRIF	
	POA= > PR= > CDIC	FOA= > PR= > CDIC	POA= > PR= > CDIC	FOA= > PR= > CDIC
	Coefficient (95% CI)	Coefficient (95% CI)	Coefficient (95% CI)	Coefficient (95% CI)
Direct effect	−0.021 (−0.039, −0.002)	0.033 (0.016, 0.050)	−0.005 (−0.018, 0.008)	0.062 (0.041, 0.083)
Mediating effect	−0.030 (−0.072, −0.003)	0.089 (0.064, 0.118)	−0.006 (−0.024, −0.001)	0.022 (0.010, 0.038)
Total effect	−0.051 (−0.072, −0.029)	0.123 (0.104, 0.142)	−0.011 (−0.024, 0.002)	0.085 (0.063, 0.106)

All coefficients are unstandardized. Confidence intervals are bias-corrected bootstrap 95% CIs. LRIF, low risk information frame; HRIF, high risk information frame; POA, present-oriented attitude; FOA, future-oriented attitude; PR, perceived risk; CDIC, cancer drugs insurance choice; CI, confidence intervals.

### *Post hoc* analysis

First, Cronbach’s *α* coefficient and KMO values of the time-orientation scale were 0.813 and 0.679 (*p* < 0.001); the corresponding results of the perceived risk scale were 0.849 and 0.865 (*p* < 0.001), indicating that both the above two scales meet our study needs. Second, results of Harman’s single-factor test showed that the largest (lowest) single factor accounted for 31.7% (18.1%) of the variation, suggesting that the CMV was not a valid threat in this study. Third, the DWH test results provided evidence of endogeneity, which may pose an issue for the regression models in the present study; however, these endogenously induced biases would be mitigated by a random block design, thus not threatening our key conclusions regarding the RIF shifting effect that we were focused. Last but not least, of the 831 eligible participants in the post-test (i.e., removed LRIF and HRIF), 52 (6.3%) did not have insured CDI. The mediating effect of perceived risk between POA and CDIC was −0.010 [95%CI: −0.025, 0.001], and between FOA and CDIC was 0.025 [95%CI: 0.012, 0.042]. Appendices D–H for details.

## Discussion

This study contributes to the existing literature by employing a randomized nationwide online survey-based experiment to explore the relative contributions of RIF to the regularity disparities of perceived risk, time orientation, and their interaction mechanism in CDI purchase decisions. Specifically, our primary finding is that the HRIF promotes CDI adoption by concurrently shifting individuals’ time orientation away from present-focus toward future-focus and increasing their perceived cancer-related risk, with perceived risk playing a mediating role.

CDI is of great significance for relieving the cancer burden in China (47). However, regarding the risk information narrative, our experimental results suggest that typical online advertisements, characterized by LRIF (see Appendix A), may be insufficient for expanding CDI coverage. Moreover, many of the health behavior interventions that have focused on how to inform people about health risks (e.g., breast cancer screening, smoking, drinking, and unhealthy eating, etc.) and then nudge them to take actions for avoiding these risks have achieved less or more desirable experimental or empirical outcomes (37, 38, 40). Under the condition of being compatible with the actual CDI schemes, these possess important enlightenment for raising the level of CDI financing and further achieving sustainable development.

In our study, the stark contrast in CDI scheme choices between LRIF and HRIF groups shows the persuasive power of meticulous, narrative-based risk information. Under HRIF, participants were significantly more likely to ensure long-term CDI schemes and less likely to forego coverage or select short-term CDI schemes. Speculating on the policy implications, marketing strategies for CDI should aim to present advertising that appears comprehensive and detailed risk information narratives. For instance, emphasizing the high incidence rate and financial burden of cancer meticulously in CDI advertising may reduce the perceived immediate costs and enhance the perceived delayed benefits associated with CDI purchase decisions.

While some scholars argued that framing risk information may not be helpful to shape individuals’ time orientation (21), we found that HRIF could simultaneously restrain POA and reinforce FOA. According to CLT, temporal distance will influence individuals to view distant future options abstractly and near future options concretely, with remote events having less psychological impact on people than immediate events due to their abstractness (48). Consequently, possible reasons may be that poor risk information may limit participants from focusing on the immediate costs of CDI, whereas meticulous risk information may allow them pay more attention to the delayed benefits of CDI. Furthermore, the post-test results with the removed RIF effect look better than those in LRIF (lower uninsured rates), probably because the crude risk information dulls individuals’ sensitivity to risk, a shift aligned with CLT.

Previous research indicates that proximal framing, linked to health risk, significantly increases risk perception of those who normally do not consider future consequences of their behaviors (21). The aforementioned research evidence and theories reveal the strong relationship between time orientation and perceived risk. Our findings further demonstrate that perceived risk, introduced as a mediating variable, could partially identify the influencing mechanism of HRIF on CDI purchase decisions. Therefore, the principle may be that when LHIF is converted to HRIF, rich and comprehensive risk information narratives restrain POA and strengthen FOA at the same time, with perceived risk mediating this effect, finally making participants more inclined to focus on purchase decisions toward insuring long-term CDI schemes. In a word, HRIF is worth considering for stimulating individuals’ CDI adoption.

Nonetheless, several limitations must be acknowledged during this study. First, self-reported decision measures and state-based assessments of time orientation and perceived risk may not fully capture real-world behavior or trait-like constructs. Second, our online sample, though nationally stratified, overrepresents younger, higher-educated individuals relative to the typical CDI target population, potentially limiting generalizability to older or less digitally engaged groups. Third, the CDI product presented was simplified and more generous than real-market policies, which may amplify willingness to insure. Fourth, the HRIF’s emotive tone, while effective in the experiment, may face regulatory or ethical constraints in actual advertising. Finally, although we controlled for key demographics, unobserved variables, such as prior insurance experience or health literacy, may still influence results. These limitations provide directions for future research endeavors.

Despite these constraints, our study underscores the potential of risk information framing to improve CDI uptake. Future research should test these mechanisms with more diverse samples, incorporate longitudinal or behavioral outcomes, and examine how real-world product complexities interact with risk communication strategies. By refining our understanding of how risk narratives shape temporal focus and risk appraisal, we can better design interventions that help individuals make informed, forward-looking health insurance choices.

## Conclusion

In recent years, attention to the impact of information frameworks on population health behavior has become an international concern. This study demonstrates that HRIF could promote individuals’ CDI adoption by weakening POA and strengthening FOA with the mediating role of perceived risk. In a world where risk information influences decision-making across every aspect of human society, many people remain trapped in unhealthy behavior patterns because of false or misleading risk information in health communication advertising. Our findings provide a reliable and practical basis for relevant authorities to formulate RIF as health interventions for CDI purchase decisions to reduce health damage and the financial burden of cancer. Especially for regulators, insurers, and health communicators, it is essential to collaboratively develop and implement evidence-based, detailed risk narratives grounded in factual statistics and relatable scenarios within CDI promotional materials to effectively guide consumer decision-making.

## Data Availability

The raw data supporting the conclusions of this article will be made available by the authors, without undue reservation.
