# Supplementary material for: The impact of risk information frameworks on cancer drug insurance (CDI) purchase decisions through time orientation and perceived risk: a survey-experiment study
Source: Front Public Health. 2026 Feb 5;14:1757999. doi: 10.3389/fpubh.2026.1757999 (PMC12916628; doi:10.3389/fpubh.2026.1757999)
Supplement: Supplementary file 1 [file Supplementary_file_1.docx]

**Appendices**

**Appendix A**

**
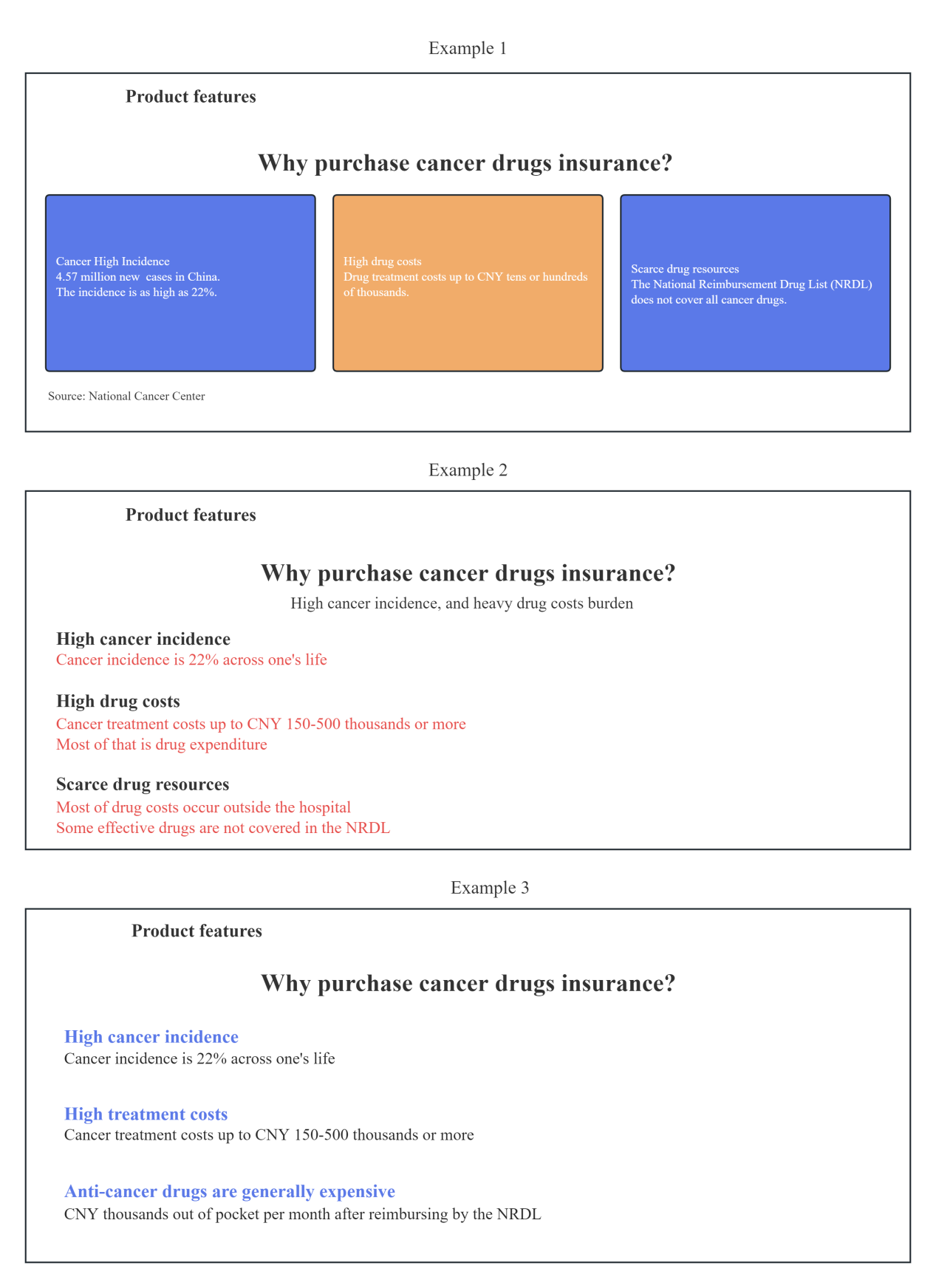
**

**Appendix B**

*LRIF (r=1, control group): National Cancer Center (NCC) presented the data showing that there are new cancer cases with 18.6% incidence rate every year in China. The National Reimbursement Drug List (NRDL) only covers a limited number of cancer drugs, and cancer drugs annual spending up to CNY tens or hundreds of thousands, imposing heavy financial burdens on most ordinary families.*

*HRIF (r=2, treatment group): National Cancer Center (NCC) presented the latest data showing that there are 4.06 million new cancer cases with 18.6% incidence rate every year, and deaths are increasing by 2.41 million per year with the incidence continues to rise in China. To date, the steep price of commercially available cancer drugs is still prohibitive. The National Reimbursement Drug List (NRDL) only covers 157 specific cancer drugs as of 2023, each pack of cancer drug that not included in NRDL costs CNY 50,000 averagely, and cancer drugs annual spending up to CNY 600,000! However, the vast majority of cancer drugs fee needs to be paid by patients themselves due to not covered by NRDL, causing great physical and mental suffering due to the lack of cancer drugs treatment and imposing heavy financial burdens on most ordinary families. This tend to mean that one person gets cancer, one or several families collapse!*

**Appendix C**

| Annual benefit limit: CNY 2 million; Deductible: CNY 0; Waiting period: 30 days  Guarantee content: 122 domestic and 60 overseas cancer drugs on the market  Guarantee duration: valid during the insurance period; Compensation ratio: 100%  Free cancer drugs provided duration after diagnosis: j years (j=1, 2, or 3), renewal not guaranteed | | |
| --- | --- | --- |
| 🞎 Option 0: Not insured | 🞎 Option 1: Just insured 1 year, premium CNY 179 | 🞎 Option 2: Insured 3 years consecutively, premium CNY 521 |

**Appendix D**

**Selected demographic characteristics of subjects for each study group (15% sample size)**

| Variable | Group 1.1 | Group 1.2 | Group 1.3 | Group 2.1 | Group 2.2 | Group 2.3 | Total | *χ^2^* | *P* |
| --- | --- | --- | --- | --- | --- | --- | --- | --- | --- |
|  | N (%) | N (%) | N (%) | N (%) | N (%) | N (%) | N (%) |  |  |
| Age |  |  |  |  |  |  |  | 9.971 | 0.822 |
| 18 to 29 years old | 73 (51.4) | 68 (49.6) | 68 (45.6) | 67 (51.1) | 76 (55.1) | 71 (53.0) | 423 (50.9) |  |  |
| 30 to 39 years old | 50 (35.2) | 51 (37.2) | 59 (39.6) | 39 (29.8) | 41 (29.7) | 39 (29.1) | 279 (33.6) |  |  |
| 40 to 49 years old | 14 (9.9) | 12 (8.8) | 15 (10.1) | 20 (15.3) | 15 (10.9) | 16 (11.9) | 92 (11.1) |  |  |
| 50 to 60 years old | 5 (3.5) | 6 (4.4) | 7 (4.7) | 5 (3.8) | 6 (4.3) | 8 (6.0) | 37 (4.5) |  |  |
| Gender |  |  |  |  |  |  |  | 5.105 | 0.403 |
| Male | 79 (55.6) | 82 (59.9) | 82 (55.0) | 67 (51.1) | 73 (52.9) | 84 (62.7) | 467 (56.2) |  |  |
| Female | 63 (44.4) | 55 (40.1) | 67 (45.0) | 64 (48.9) | 65 (47.1) | 50 (37.3) | 364 (43.8) |  |  |
| Marital |  |  |  |  |  |  |  | 5.447 | 0.364 |
| Married | 63 (44.4) | 72 (52.6) | 85 (57.0) | 70 (53.4) | 67 (48.6) | 69 (51.5) | 426 (51.3) |  |  |
| Not married | 79 (55.6) | 65 (47.4) | 64 (43.0) | 61 (46.6) | 71 (51.4) | 65 (48.5) | 405 (48.7) |  |  |
| Residence |  |  |  |  |  |  |  | 7.714 | 0.173 |
| Rural | 41 (28.9) | 52 (38.0) | 46 (30.9) | 38 (29.0) | 54 (39.1) | 52 (38.8) | 283 (34.1) |  |  |
| Urban | 101 (69.6) | 85 (65.7) | 103 (66.6) | 93 (68.6) | 84 (65.4) | 82 (64.8) | 548 (66.8) |  |  |
| Annual disposable income individually |  |  |  |  |  |  |  | 14.622 | 0.479 |
| Up to 19999 CNY | 30 (21.1) | 28 (20.4) | 32 (21.5) | 30 (22.9) | 38 (27.5) | 22 (16.4) | 180 (21.7) |  |  |
| 20000 to 29999 CNY | 12 (8.5) | 15 (10.9) | 8 (5.4) | 13 (9.9) | 15 (10.9) | 13 (9.7) | 76 (9.1) |  |  |
| 30000 to 49999 CNY | 57 (40.1) | 59 (43.1) | 63 (42.3) | 61 (46.6) | 51 (37.0) | 66 (49.3) | 357 (43.0) |  |  |
| At least 50000 CNY | 43 (30.3) | 35 (25.5) | 46 (30.9) | 27 (20.6) | 34 (24.6) | 33 (24.6) | 218 (26.2) |  |  |
| Education |  |  |  |  |  |  |  | 16.671 | 0.674 |
| Junior | 5 (3.5) | 2 (1.5) | 5 (3.4) | 3 (2.3) | 4 (2.9) | 5 (3.7) | 24 (2.9) |  |  |
| Senior | 18 (12.7) | 23 (16.8) | 19 (12.8) | 21 (16.0) | 16 (11.6) | 22 (16.4) | 119 (14.3) |  |  |
| Associate | 47 (33.1) | 32 (23.4) | 28 (18.8) | 33 (25.2) | 36 (26.1) | 37 (27.6) | 213 (25.6) |  |  |
| Undergraduate | 65 (45.8) | 69 (50.4) | 88 (59.1) | 69 (52.7) | 74 (53.6) | 61 (45.5) | 426 (51.3) |  |  |
| Postgraduate | 7 (4.9) | 11 (8.0) | 9 (6.0) | 5 (3.8) | 8 (5.8) | 9 (6.7) | 49 (5.9) |  |  |
| Health insurance type |  |  |  |  |  |  |  | 16.285 | 0.699 |
| Free | 15 (10.6) | 14 (10.2) | 16 (10.7) | 17 (13.0) | 10 (7.2) | 18 (13.4) | 90 (10.8) |  |  |
| Urban employees | 57 (40.1) | 51 (37.2) | 66 (44.3) | 52 (39.7) | 50 (36.2) | 44 (32.8) | 320 (38.5) |  |  |
| Urban and rural residents | 57 (40.1) | 59 (43.1) | 59 (39.6) | 58 (44.3) | 68 (49.3) | 63 (47.0) | 364 (43.8) |  |  |
| Commercial | 9 (6.3) | 7 (5.1) | 6 (4.0) | 2 (1.5) | 6 (4.3) | 7 (5.2) | 37 (4.5) |  |  |
| Without | 4 (2.8) | 6 (4.4) | 2 (1.3) | 2 (1.5) | 4 (2.9) | 2 (1.5) | 20 (2.4) |  |  |
| Sample size | 142 | 137 | 149 | 131 | 138 | 134 | 831 |  |  |

**Appendix E**

**Results of purchasing decisions for CDI schemes (15% sample size)**

| Framework | Group | Option 0 | Option 1 | Option 2 | *χ^2^* | *P* |
| --- | --- | --- | --- | --- | --- | --- |
|  |  | Number (%) | Number (%) | Number (%) |  |  |
| LRIF | 1.1 | 7 (4.9) | 49 (34.5) | 86 (60.6) | 3.866 | 0.145 |
|  | 1.2 | 14 (10.2) | 40 (29.2) | 83 (60.6) | 4.895 | 0.087 |
|  | 1.3 | 8 (5.4) | 27 (18.1) | 114 (76.5) | 9.621 | 0.008 |
| HRIF | 2.1 | 7 (5.3) | 38 (29.0) | 86 (65.6) | 0.274 | 0.872 |
|  | 2.2 | 7 (5.1) | 41 (29.7) | 90 (65.2) | 0.571 | 0.752 |
|  | 2.3 | 9 (6.7) | 37 (27.6) | 88 (65.7) | 0.060 | 0.971 |
| *χ^2^* | | 4.858 | 10.586 | 11.008 |  |  |
| *P* | | 0.434 | 0.060 | 0.051 |  |  |

Abbreviations: LRIF=low risk information framework; HRIF=high risk information framework; Option 0=not insured; Option 1=just insured 1 year, 179 CNY premium; Option 2=insured 3 years consecutively, 179 CNY premium.

**Appendix F**

**Results of time orientation towards CDI purchasing (15% sample size)**

| Dimensions | Items | LRIF (N=428) | HRIF (N=403) | Comparison | |
| --- | --- | --- | --- | --- | --- |
|  |  | M (SD) | M (SD) | t (829) | *P* |
| Present-oriented | I focus on my current NOT future cancer risk when I choose the CDI scheme | 3.70 (1.060) | 3.83 (0.998) | 1.822 | 0.092 |
|  | I care more about current premium NOT future cost reduce when I choose the CDI scheme | 3.81 (0.994) | 3.82 (0.939) | 0.149 | 0.119 |
| Future-oriented | I value more on the future security when I choose the CDI scheme | 3.95 (0.948) | 3.97 (0.902) | 0.260 | 0.166 |
|  | I consider its long-term impact on my future life seriously when I choose the CDI scheme | 4.06 (0.872) | 4.08 (0.803) | 0.366 | 0.402 |

Abbreviations: LRIF=low risk information framework; HRIF=high risk information framework

**Appendix G**

**Results of perceived risk related to developing cancer (15% sample size)**

| Dimensions | Items | LRIF (N=428) | HRIF (N=403) | Comparison | |
| --- | --- | --- | --- | --- | --- |
|  |  | M (SD) | M (SD) | t (5581) | *P* |
| Cancer | Carcinogenic factors are ubiquitous in daily living and working conditions | 4.12 (0.922) | 4.07 (0.929) | 0.735 | 0.710 |
|  | I'm worried about developing cancer in my life | 3.82 (1.050) | 3.86 (0.997) | 0.506 | 0.066 |
| Financial | I'm worried about the price of cancer drugs is steep | 2.75 (1.400) | 2.76 (1.322) | 0.176 | 0.057 |
|  | If I get cancer, the cancer drugs expenses are unaffordable for my family | 3.96 (1.062) | 4.05 (0.941) | 1.350 | 0.077 |
|  | Cancer drugs insurance could avoid economic risk caused by cancer | 3.93 (0.946) | 3.97 (0.929) | 0.586 | 0.682 |

Abbreviations: LRIF=low risk information framework; HRIF=high risk information framework

**Appendix G**

The Mediating Effect of Perceived Risk between Time Orientation and CDI Purchase Decisions (15% sample size)

| **Variables** | **No-RIF** | |
| --- | --- | --- |
|  | **POA=>PR=>CDIC** | **FOA=>PR=>CDIC** |
|  | **Coefficient ( 95% CI)** | **Coefficient (95% CI)** |
| **Direct effect** | -0.035 (-0.055, -0.015) | 0.050 (0.031, 0.070) |
| **Mediating effect** | -0.010 (-0.025, 0.001) | 0.025 (0.012, 0.042) |
| **Total effect** | -0.045 (-0.065, -0.025) | 0.075 (0.055, 0.095) |

**Notes:** All coefficients are unstandardized. Confidence intervals are bias-corrected bootstrap 95% CIs. LRIF=low risk information frame; HRIF=high risk information frame; POA=present-oriented attitude; FOA=future-oriented attitude; PR=perceived risk; CDIC=cancer drugs insurance choice; CI=confidence intervals.
